# Selection of Suitable Reference Genes for RT-qPCR Gene Expression Analysis in Centipedegrass under Different Abiotic Stress

**DOI:** 10.3390/genes14101874

**Published:** 2023-09-26

**Authors:** Xiaoyun Wang, Xin Shu, Xiaoli Su, Yanli Xiong, Yi Xiong, Minli Chen, Qi Tong, Xiao Ma, Jianbo Zhang, Junming Zhao

**Affiliations:** 1 College of Grassland Science and Technology, Sichuan Agricultural University, Wenjiang, Chengdu 611130, Chinayanlimaster@126.com (Y.X.);; 2 Sichuan Academy of Grassland Sciences, Pidu, Chengdu 611731, China; chenlimin1986816@126.com (M.C.); djyqq@126.com (Q.T.)

**Keywords:** abiotic stress, centipedegrass, reference genes, RT-qPCR

## Abstract

As a C4 warm-season turfgrass, centipedegrass (*Eremochloa ophiuroides* (Munro) Hack.) is known for its exceptional resilience to intensive maintenance practices. In this research, the most stably expressed reference genes in the leaves of centipedegrass under different stress treatments, including salt, cold, drought, aluminum (Al), and herbicide, were screened by the quantitative real-time PCR (RT-qPCR) technique. The stability of 13 candidate reference genes was evaluated by software GeNorm V3.4, NormFinder V20, BestKeeper V1.0, and ReFinder V1.0. The results of this experiment demonstrated that the expression of the *UBC* (*ubiquitin-conjugating enzyme*) remained the most stable under cold and Al stress conditions. On the other hand, the *MD* (*malate dehydrogenase*) gene exhibited the best performance in leaf tissues subjected to salt and drought stresses. Under herbicide stress, the expression level of the *RIP* (*60S ribosomal protein L2*) gene ranked the highest. The expression levels of abiotic stress-associated genes such as *PIP1*, *PAL*, *COR413*, *ALMT9*, and *BAR* were assessed to validate the reliability of the selected reference genes. This study provides valuable information and reference points for gene expression under abiotic stress conditions in centipedegrass.

## 1. Introduction

Due to its excellent repeatability, strong specificity, and high throughput, quantitative real-time PCR (RT-qPCR) has become the principal method employed for gene expression analysis [1,2,3,4]. This technique plays a crucial role in investigating plant gene expression patterns under various conditions. Traditionally, certain housekeeping genes like *ACT*, *TUB*, *UBC*, etc., are commonly employed as reference genes [5,6,7]. However, it is important to note that the expression levels of these genes may vary under various experimental settings. Based on previous studies, the expression of reference genes can vary across different experimental conditions, including various growth stages, tissues, and abiotic stresses [8]. Hence, it is crucial to identify reference genes with specific expression patterns under distinct experimental conditions to ensure accurate quantification of expression levels in response to diverse abiotic stressors. So far, reference genes have been studied and reported in numerous plant species, such as *Arabidopsis thaliana* [9], rice (*Oryza sativa*) [10], *Setaria viridis* [11], and Kentucky bluegrass (*Poa pratensis*) [12], etc.

Centipedegrass (*Eremochloa ophiuroides*) is a diploid warm-season perennial grass species (2n = 2x = 18) belonging to the genus *Eremochloa* in the Poaceae family [13]. Owing to its low fertility requirements and minimal management needs, centipedegrass is commonly referred to lazy grass. It is native to the subtropical region of central and southern China and is primarily distributed in various provinces and regions located south of the Yangtze River Basin [14,15,16]. The stolons of centipedegrass are well-developed and robust, enabling strong and rapid spreading. It naturally occurs in humid grasslands and along riverbanks and is commonly found in fertile and moist soil areas like forest edges and valley slopes. It is an ideal turfgrass species for establishing broad-leaved lawns [17,18]. Additionally, centipedegrass is highly valuable for soil reinforcement, slope protection, and green construction. It possesses extensive applications in the establishment of green lawns, soil and water conservation efforts, as well as highway slope landscaping projects, both domestically and internationally [19]. However, compared to other warm-season turfgrasses, centipedegrass is primarily constrained by its limited salt tolerance [20]. Moreover, as a warm-season turfgrass, centipedegrass is highly vulnerable to low temperature stress, which significantly impedes its widespread adoption and utilization in temperate and warm temperate regions. Furthermore, due to the increasing severity of soil acidification, aluminum (Al) toxicity is the main cause of harm to plants in acidic soils. Studies have shown that plant root tips respond to micromolar concentrations of Al, leading to rapid inhibition of root growth and damage to root morphology, which has also been reported in centipedegrass [18]. Thus, identifying internally stable expressed reference genes under Al stress in centipedegrass is of great importance for future breeding efforts. Compared to crops, centipedegrass is considered a weed that affects crop yield. Hence, herbicides are commonly used to control weeds and improve crop production. However, prolonged herbicide use can lead to the development of resistance in plants. Interestingly, research has shown that herbicide resistance evolution is extremely rare in grass species [21]. As centipedegrass is commonly used as a turfgrass, identifying plants with herbicide resistance is of significant importance for turfgrass breeding. Therefore, comprehending the expression patterns of genes involved in abiotic stress response is crucial for elucidating the molecular mechanisms of how centipedegrass responds to stress and enhancing its adaptability under stressful conditions.

Recent studies have identified a set of exceptional reference genes that are expressed in various stress conditions and plant organs [22,23,24]. However, no research has yet been carried out on the screening of reference genes in centipedegrass under different abiotic stresses. The aim of this study was to identify and select the most stable reference genes across a range of abiotic stress conditions by considering 13 commonly employed housekeeping genes as potential genes. Meanwhile, we employed GeNorm [25], NormFinder [26], BestKeeper [27], and RefFinder [28] to identify the ideal reference genes with optimal performance under different stresses. Finally, we validated these selected candidate reference genes by utilizing the target genes *plasma membrane intrinsic protein 1* (*PIP1*), *phenylalanine ammonia-lyase* (*PAL*), *cold-regulated gene 413* (*COR413*), *aluminum-activated malate transporter 9* (*ALMT9*), and *bialaphos resistance* (*BAR*).

## 2. Materials and Methods

### 2.1. Material Culture and Stress Treatment

The seeds of centipedegrass variety ‘Wuling’ were soaked in a 10% NaOH solution for 5 min with continuous stirring. After treatment, the seeds were washed with distilled water. Subsequently, 1.0 g of seeds was sown in a small square pot filled with quartz sand and cultured in a plant growth chamber. The culture conditions were as follows: temperature 23 °C/19 °C (12 h day/12 h night), photoperiod 12 h, culture for 90 days for subsequent experiments. Drought stress was simulated using 20% PEG-6000, 200 mmol·L^−1^ NaCl solution was used for salt stress, cold stress was carried out in 4 °C incubator, 100 μmol·L^−1^ AlCl_3_ solution was used for Al stress, and 6 mmol·L^−1^ glufosinate was sprayed on leaves under herbicide stress, and the control plants were irrigated with the same amount of Hoagland nutrient solution. Each treatment was set with three replicates. Samples were taken at 0, 0.5, 1.5, 3, 6, 12, 24, 48, and 72 h after stress treatment. After sampling, it is necessary to quickly freeze the samples in liquid nitrogen and store them at −80 °C in an ultra-low temperature environment for future use.

### 2.2. RNA Extraction and Reverse Transcription

Tissuelyzer (Qiagen, Germantown, MD, USA) was used to crush 50–100 mg centipedegrass leaves. Total RNA extraction was carried out with the M5 HiPer Plant Complex Mini Kit (Juhemei, Beijing, China), yielding a concentration of 200 ng. RNA was then reverse transcribed into cDNA using the M5 Super plus qPCR RT kit and gDNA Remover (Juhemei, Beijing, China). It was stored at −20 °C for further use.

### 2.3. Designing and Validating Primers with Specificity

Thirteen reference genes, including ubiquitin-conjugating enzyme (*UBC*), glyceraldehyde-3-phosphate dehydrogenase (*GADPH*), actin (*ACT*), sucrose synthase (*SuS*), alkaline and neutral invertase (*ANI*), ADP-ribosylation factor (*ADP*), cyclophilin (*CYP*), histone H3 (*H3*), 50S ribosomal protein L2 (*50S*), 60S ribosomal protein L2 (*RIP*), malate dehydrogenase (*MD*), chaperone protein (*CP*), and heat shock 70 kDa protein (*HSP70*), were chosen as candidate reference genes based on the transcriptome data obtained from centipedegrass [17]. We used the online software Primer Quest for primer design (https://sg.idtdna.com/pages/tools/primerquest, accessed on 15 January 2023), and the primers were synthesized by Youkang Biotech (Chengdu, China) (Table S1). The specificity of primers was verified by RT-qPCR reaction dissolution curve.

### 2.4. Real-Time Quantitative PCR

RT-qPCR was performed using 2× M5 Hi Per SYBR Premix Es Taq reagent (Juhemei, Beijing, China) and CFX96 Realtime PCR system (Bio-Rad, Hercules, CA, USA). The experiment was carried out in a 10 μL system and ice bath. The reaction system was as follows: primer 0.4 μL, 2× M5 HiPer SYBR Premix Es Taq 5 μL, cDNA 1 μL, and finally supplemented with ddH_2_O to 10 μL. The experimental protocol commenced with an initial denaturation phase at 95 °C for a duration of 10 min. Subsequently, 35 cycles of denaturation were performed at 95 °C for 15 s, by annealing at 55 °C for a duration of 1 min. Finally, a final extension step was carried out. Each sample was analyzed with three biological and three technical replicates.

### 2.5. Data Analysis and Stability Ranking

Firstly, the cycle threshold (Ct) values for the reference genes were obtained using RT-qPCR. Subsequently, the reference genes were comprehensively analyzed and ranked using GeNorm, NormFinder, BestKeeper, and RefFinder (http://blooge.cn/RefFinder/?type=reference, accessed on 15 February 2023). Typically, when analyzing the data with GeNorm and NormFinder, Ct values are converted into relative quantification values (2^−ΔCt^) (ΔCt = Ct_sample_ − Ct_min_. Here, Ct_sample_ represents the Ct value of the housekeeping gene, while Ct_min_ represents the lowest Ct value observed for the housekeeping gene under each abiotic stress condition), and each candidate reference gene is sorted accordingly. Moreover, the GeNorm software is capable of calculating the pairwise variation V_n_/V_n+1_. Pairwise variation compares the variation between the normalization coefficients calculated from two reference genes with the variation from their geometric mean. The lower pairwise variation value indicates that the normalized factor is more stable, so less reference genes are required. If the V_n_/V_n+1_ ratio is below 0.15, it suggests that there is no need to include additional reference genes, that is, n is the ideal number of reference genes [29]. BestKeeper directly calculates the standard deviation (SD) and coefficient of variation (CV) from the Ct values to perform ranking. Finally, RefFinder combines the above three calculation methods to comprehensively sort the reference genes.

## 3. Results

### 3.1. Identification of the Primer Specificity

The RT-qPCR reaction was performed using cDNA from centipedegrass leaves under different abiotic stress conditions as templates. Based on the results, the melting curves of all candidate reference genes exhibited a unimodal pattern under the five different abiotic stress conditions (Figure S1). Additionally, the amplification curves demonstrated excellent reproducibility, confirming that the designed primers specifically amplified the desired gene products without any primer dimer formations and can be used for further study.

### 3.2. Analysis of Reference Gene Expression

**Figure 1:**
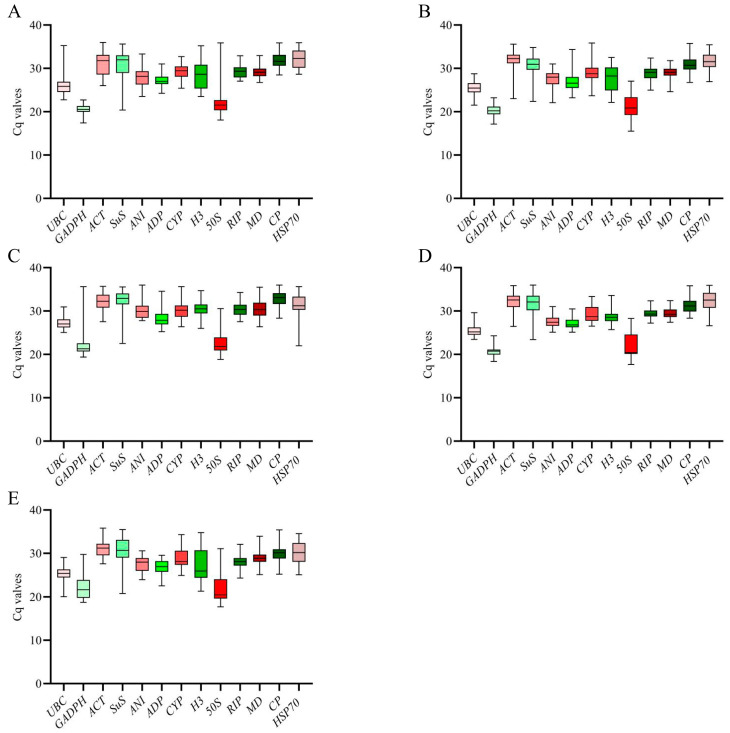
The expression levels of the thirteen candidate reference genes were assessed under abiotic stress conditions. On the *y*-axis, the cycle threshold (Ct) values of these thirteen candidate reference genes in all samples were plotted. A horizontal line within the box plot graph represents the median Ct value. The lower and upper bounds of the boxes represent the 25th and 75th percentiles, respectively, while the whiskers indicate the range across all samples. (**A**) Drought stress, (**B**) Salt stress, (**C**) Cold stress, (**D**) Al stress, (**E**) Herbicide stress.

According to previous studies, the Ct value is an inverse indicator of gene expression, where a higher Ct value corresponds to lower levels of gene expression [30]. We analyzed the expression levels of 13 reference genes using RT-qPCR (Figure 1). The results revealed a range of Ct values, varying from 17.4 to 35.99, for the reference genes. The Ct values of *GADPH* under salt, drought, and Al stress were the lowest, which were 17.15, 17.4, and 18.38, respectively, which suggests that the abundance of *GADPH* expression was the highest.

### 3.3. Assessment of Expression Stability of Candidate Reference Genes

#### 3.3.1. GeNorm Analysis

**Figure 2:**
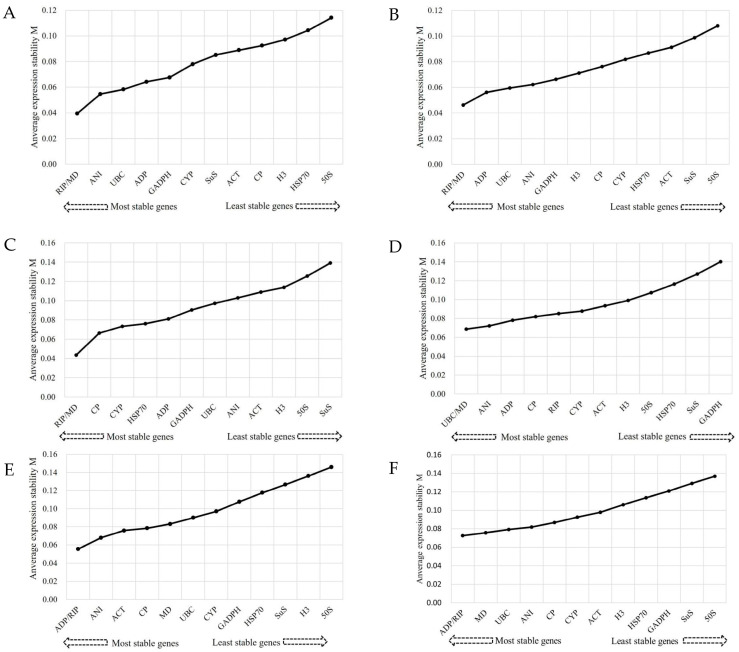
Expression stability measurement (M) for thirteen candidate reference genes under abiotic stress conditions. (**A**) Salt stress, (**B**) Al stress, (**C**) Drought stress, (**D**) Cold stress, (**E**) Herbicide stress, (**F**) All Samples.

In GeNorm analysis, the consistency of gene expression is typically assessed using the M value. Generally speaking, the M value represents the degree of stability in expression levels and is negatively correlated with the expression level of the reference gene. In all abiotic stress samples, *ADP* and *RIP* were identified as the genes with the highest level of stability, while *50S* displayed the least consistent expression. Under salt, drought, and Al stress conditions, *RIP* and *MD* were found to be the most stable genes, whereas *UBC* and *MD* exhibited the highest reliability under cold stress. In the case of herbicide treatment, the *ADP* and *RIP* genes demonstrated the highest stability in expression; however, *50S* was the least stable (Figure 2).

**Figure 3:**
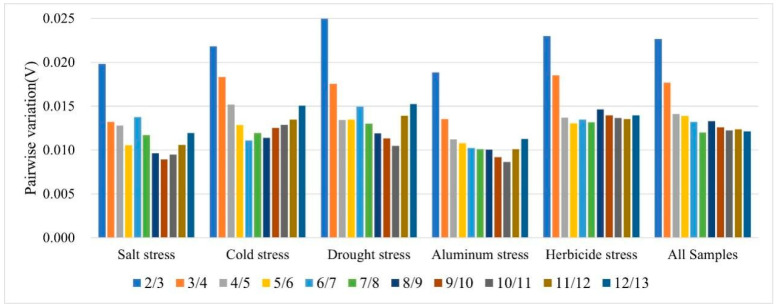
GeNorm software was utilized to calculate the pairwise variation (V) of the candidate reference genes. The V_n_/V_n+1_ values derived from this analysis were then utilized to determine the optimal number of reference genes (n).

The common practice in determining the optimal number of reference genes for normalizing target gene expression involves the use of pairwise variation analysis (V_n_/V_n+1_) [31,32]. If the V_n_/V_n+1_ value is below 0.15, it suggests that two stable reference genes are sufficient to meet the quantitative correction under the corresponding conditions. The findings from this study demonstrated that the V_2_/V_3_ ratios for each reference gene across all samples under various abiotic stress conditions were consistently below the 0.15 threshold (Figure 3); they showed that two reference genes were needed to attain the optimal performance of gene expression analysis.

#### 3.3.2. BestKeeper Analysis

BestKeeper is a widely utilized software for assessing the stability of expression levels among candidate reference genes [33]. The stability of each candidate reference gene was assessed through the calculation of both the coefficient of variation (CV) and standard deviation (SD). The stability of a reference gene is considered better if it has a lower CV ± SD value [34]. In our study, the expression of *RIP* exhibited the optimal stability under Al and herbicide stress, with the lowest CV ± SD values of 3.1 ± 0.91 and 3.83 ± 1.07, respectively. Furthermore, *UBC* and *MD* were recognized as the genes displaying the most consistent expression patterns in response to cold, salt, and drought stress. Among the chosen reference genes, *MD* achieved the highest ranking (Table 1).

#### 3.3.3. NormFinder Analysis

NormFinder is a widely employed computational tool for evaluating the stability of reference gene expression. It aids in the determination of the most suitable reference gene by evaluating its expression stability, with the lowest value indicating the highest level of stability [35]. The results revealed that *UBC* exhibited the highest reliability under cold stress; *MD* demonstrated the most stability under salt stress, drought, and Al stress; and *ANI* was the most stable under herbicide stress. Conversely, *HSP70* showed the least stability under salt, cold, and drought stresses. Overall analysis of all samples indicated that *MD* was the most reliable gene, followed by *RIP* and *ADP* (Table 2).

#### 3.3.4. ReFinder Analysis

ReFinder is an integrated analysis tool used for the validation of reference genes. It incorporates several methods including GeNorm, BestKeeper, NormFinder, and delta Ct analysis [36]. In our study, ReFinder analysis revealed that *UBC* and *MD* exhibited the highest stability in gene expression under cold and Al stress conditions. Under salt and drought stress conditions, *MD* and *RIP* emerged as the genes with the highest stability. Additionally, *RIP* and *ANI* exhibited the best expression stability under herbicide stress. Among all the samples, *ANI* and *RIP* were identified as the most reliable genes in the comprehensive analysis. Conversely, *50S*, *CP*, and *HSP70* were found to be the three least consistent reference genes (Table 3).

#### 3.3.5. Verification of the Screened Reference Genes

**Figure 4:**
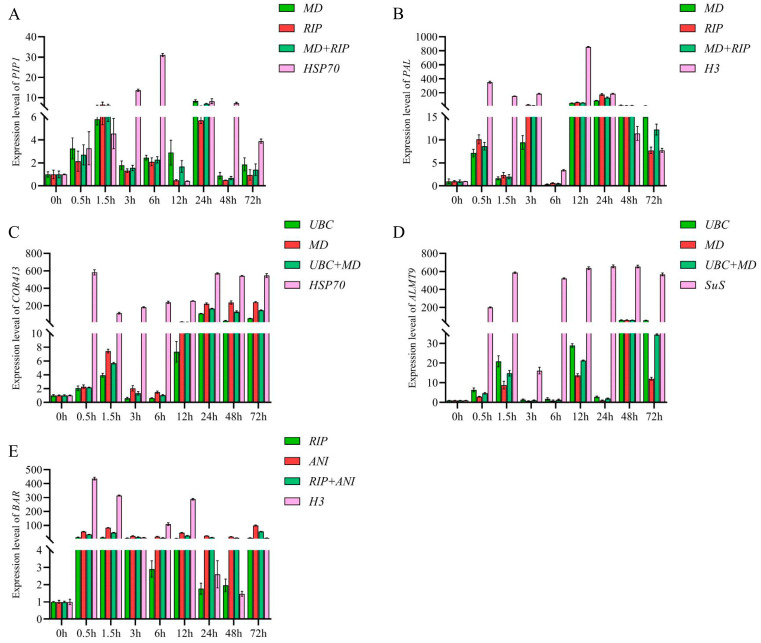
Expression levels of target genes in centipedegrass under different abiotic stresses at different times. Bars indicate standard errors. (**A**) Drought stress, (**B**) Salt stress, (**C**) Cold stress, (**D**) Al stress, (**E**) Herbicide stress.

Based on our above findings, five genes (*PAL*, *PIP1*, *COR413*, *ALMT9*, and *BAR*) that were widely known for their important roles in plant stress resistance [37,38,39,40,41] were used to analyzed their expression patterns under various stress treatments with the most reliable and least reliable combination of candidate reference genes. Under drought stress, the *PIP1* gene was selected for verification. When normalizing the data using the combination of *MD* and *RIP*, the relative expression level of the target gene showed consistent trends. In order to normalize the expression of *PIP1*, the *HSP70* gene, which had the lowest ranking, was used as a reference; its expression level reached its peak at 6 h of stress, being approximately 30 times higher than at 0 h. This result was contradictory to the expression trends observed for the other reference genes (Figure 4A). During salt stress, the *PAL* gene was chosen for verification. Upon normalization, using the most stable combination of genes (*MD* and *RIP*), the expression level of *PAL* exhibited an initial increase followed by a subsequent decrease. Nonetheless, utilizing the *H3* gene for normalization resulted in the attainment of the maximum expression of *PAL* at 12 h, followed by a gradual decline (Figure 4B). Under cold stress, the *COR413* gene was selected for verification. When the expression of target gene was normalized using the combination of genes with the highest stability (*UBC* and *MD*), the expression of *COR413* gene exhibited an initial increase, followed by a decrease, and then a gradual increase. However, when normalized using the most unstable gene, *HSP70*, there was no significant trend observed in the expression of *COR413* (Figure 4C). Under Al stress, the *ALMT9* gene was selected for verification. When normalized using the *SuS* gene, the expression trend of *ALMT9* did not change significantly, and the expression level remained extremely high. However, when normalized using the most reliable expressed gene (*UBC* or *MD*), the expression levels of *ALMT9* were lower (Figure 4D). Under herbicide stress, the *BAR* gene was selected for verification. When normalized using the combination of the most reliable reference genes (*RIP* and *ANI*), the expression of *BAR* initially decreased and then increased. At 72 h, the expression of the *BAR* gene reached its maximum. However, the normalization of *BAR* expression using the *H3* gene demonstrated a decreasing trend, which contrasted with the expression pattern observed with the most optimal reference gene (Figure 4E).

## 4. Discussion

As a reference for normal cell function, reference genes can help to identify changes in gene expression under different conditions and related functional regulation mechanisms, thus deepening the understanding of various physiological processes in organisms [42]. However, it is important to note that housekeeping genes can exhibit specific differential expression patterns depending on the species, tissue types, and experimental conditions [43]. For example, in celery (*Apium graveolens*) under heat stress, *GADPH* ranked second in terms of stability [44], but under mulberry (*Fructus Mori*) cold stress, it was found to be the most unstable [45]. In blueberries (*Vaccinium uliginosum*) subjected to salt stress, the stably expressed gene was *PP2A*, and under drought stress, *TBP* expression was found to be the most stable [46]. Previous research has provided evidence that the stability of reference genes in plants can vary across different tissues. For example, under salt stress, *GAPD* was identified as the most reliable reference gene in oat (*Avena sativa*) leaves, while *ADPR* exhibited the most stable expression as a reference gene in the roots [47]. After heat stress, it was found that *SAND* and *F-box* were stably expressed in leaf tissues of winter rapeseed (*Brassica rapa* L.); among the stable reference genes in roots, *PP2A* and *RPL* exhibited the highest stability [48]. Hence, the identification of reference genes with stable expression under specific conditions is of utmost importance.

To enhance the precision of the analysis outcomes, this study utilized three frequently employed software (BestKeeper, NormFinder, and GeNorm) to comprehensively rank the stability of 13 reference genes under five abiotic stresses. When using GeNorm software for analysis, the principle is to convert the Ct value of the reference gene into a logarithmic value (2^−ΔCt^), calculate the *M* value [32], and subsequently rank the stability. Simultaneously, the software allows for the determination of the optimal quantity of reference genes for quantitative analysis: when the pairwise coefficient of variation (V_n_/V_n+1_) < 0.15, the number of reference genes indicated by the n value is the most suitable. The results of this study showed that two reference genes were entailed for gene expression analysis to achieve the best performance. It is worth noting that BestKeeper differs from other software rankings, as it can significantly enhance the ranking of a specific gene [27]. For example, when exposed to drought stress, BestKeeper revealed *GADPH* as the most stable gene, whereas NormFinder identified *GADPH* as the fourth-ranked gene, which is consistent with previous research findings [49,50,51]. NormFinder determines gene expression stability by calculating 2^−ΔCt^. However, due to the use of different calculation methods by these software, there may be variations in the stability ranking of reference genes [52]. For example, in this study, under drought stress conditions, both NormFinder and GeNorm identified the *MD* gene as the most stable gene, while BestKeeper indicated *GADPH* as the most stable gene, which has been previously reported [53]. RefFinder combines the algorithms of the three software to integrate the candidate reference genes and generate a composite ranking, so the results of RefFinder show the overall ranking [36]. In this study, *UBC* demonstrated the highest stability under cold and Al stress conditions. Furthermore, *UBC* has been consistently reported as the top reference gene in other studies, such as the case of *Cryptomeria fortunei*, where *UBC* exhibited the most stable expression under GA3 (gibberellic acid 3) and MeJA (methyl jasmonate) hormone treatment [54]. *RIP* is considered to be the most reliable reference gene under herbicide stress. Similarly, *RIP* has been identified as the most consistently expressed gene in root tissues under salt stress and in leaf tissues under drought stress in winter rapeseed [48], while *RIP* is ranked lower under abiotic stress in mulberry [45] and *Cryptomeria fortune* [54]. This is mainly due to different plant tissues and different organs, which will affect the stability of the reference gene.

According to previous studies, genes belonging to the same family also exhibit diverse expression patterns across various developmental stages, tissues, or under different stress conditions. For example, in the root of Brachiaria grass (*Brachiaria mutica*) under salt stress, the *ACT12* gene ranks second in terms of stability [6]. However, under salt stress in *Suaeda glauca*, *ACT7* shows lower stability [55]. In this study, *50S* and *RIP* genes belong to the same gene family. It is observed that *RIP* genes consistently rank higher under each stress condition, while *50S* genes rank lower. Therefore, under the same experimental conditions, the stability in expression of reference genes may vary across different species. Furthermore, it is important to note that genes within the same family may exhibit variable expression patterns under different conditions [56]. Heat shock proteins (Hsps) are a class of proteins that are usually formed and expressed under certain environmental factors or stress conditions [57,58]. *HSP70* is an extensively conserved stress-inducible protein, classified within the heat shock protein family. As a molecular chaperone, it plays a pivotal role in cellular defense against diverse extreme stressors, such as heat and oxidative stress [59]. Therefore, *HSP70* has significant physiological importance in protecting cells from stress-induced damage. For example, the level of expression for *HSP70* exceeds that observed in heat-sensitive genotypes, indicating its superior protective effect against heat stress [60], However, its expression level may not be well reflected under other stress conditions. Furthermore, the expression of *HSP70* was unstable under herbicide stress of Johnsongrass (*Sorghum halepense*) [7], and similar results were found in this study. Upon analyzing all samples, the expression of *HSP70* was found to be the most unstable, and this pattern was consistent under both cold and drought stress. This indicates the potential variation in the expression of *HSP70* in response to different conditions. Therefore, it is essential to consider specific circumstances when selecting reliable reference genes.

According to the current research, many genes involved in plant abiotic stress have been found. These genes were highly significant regulating plant stress resistance. For instance, the aquaporin water channel gene (*PIP1*) plays a role in regulating stomatal activity [38,61]. Additionally, the *BAR* gene is involved in modifying and inhibiting the activity of plant glutamine synthetase, thereby facilitating weed control [41,62]. Furthermore, the cold-adapted protein *COR413,* which responds to low temperature stress, has been extensively investigated and applied [39,63]. Therefore, to confirm the stability of reference gene expression, further validation was performed, and we selected *PAL*, *PIP1, COR413*, *ALMT9* and *BAR* as the target genes under salt, drought, cold, Al, and herbicide stress, respectively. Based on previous studies, it is recommended to assess the relative expression level of the target gene using a minimum of two stable reference genes or more [64,65]. Therefore, two genes with excellent expression levels and a high comprehensive ranking were selected, along with the gene with the poorest expression level, to investigate the expression patterns of the target gene. Through gene expression verification, it was observed that the expression pattern of the target gene remained consistent when utilizing a combination of stable reference genes for normalization. However, deviation from stable expression results of the reference genes was observed when the reference gene with the expression stability was used for normalization analysis. These observations affirm the reliability of the identified reference genes.

## 5. Conclusions

In this study, the reference genes of centipedegrass under five different abiotic stresses were screened for the first time. The experimental results indicated that the *UBC* gene demonstrated optimal performance under cold and Al stress conditions, while the *MD* gene was determined to be the most appropriate reference gene under drought and salt stress. Additionally, the *RIP* gene ranked first under herbicide stress. Under cold and drought stress, *HSP70* exhibited the highest level of instability. *H3* displayed the most instability under saline conditions and herbicide stress, while *SuS* ranked last under Al stress. The findings of this study serve as a basis and point of reference for future investigations into gene expression analysis. Furthermore, these discoveries carry significant importance in unraveling the molecular mechanisms that underlie centipedegrass response to abiotic stress.

## Figures and Tables

**Table 1:** BestKeeper software was utilized to calculate the expression stability values of candidate reference genes in centipedegrass.

Rank	Cold Stress	Salt Stress	Drought Stress	Al Stress	Herbicide Stress	All Samples
1	*UBC*(4.24 ± 1.15)	*MD*(3.63 ± 1.05)	*GADPH*(5.08 ± 1.04)	*RIP*(3.1 ± 0.91)	*RIP*(3.83 ± 1.07)	*MD*(4.26 ± 1.25)
2	*RIP*(4.3 ± 1.31)	*GADPH*(5.68 ± 1.15)	*MD*(3.66 ± 1.07)	*GADPH*(4.65 ± 0.97)	*ADP*(4.79 ± 1.29)	*ADP*(4.79 ± 1.3)
3	*CP*(4.09 ± 1.35)	*ADP*(4.99 ± 1.32)	*ADP*(4.38 ± 1.19)	*MD*(3.36 ± 0.99)	*MD*(4.62 ± 1.34)	*RIP*(4.52 ± 1.32)
4	*H3*(4.72 ± 1.44)	*UBC*(5.33 ± 1.35)	*RIP*(4.25 ± 1.25)	*UBC*(4.09 ± 1.05)	*UBC*(5.39 ± 1.35)	*UBC*(5.37 ± 1.39)
5	*ADP*(5.12 ± 1.45)	*RIP*(4.7 ± 1.07)	*CYP*(5.02 ± 1.46)	*ADP*(3.93 ± 1.07)	*ACT*(4.68 ± 1.45)	*GADPH*(7.4 ± 1.57)
6	*MD*(4.95 ± 1.51)	*CP*(4.39 ± 1.36)	*CP*(4.79 ± 1.52)	*ANI*(4.08 ± 1.12)	*ANI*(5.53 ± 1.52)	*CP*(5.34 ± 1.67)
7	*ACT*(4.74 ± 1.52)	*HSP70*(5.32 ± 1.68)	*UBC*(5.9 ± 1.54)	*H3*(4.32 ± 1.24)	*CP*(5.52 ± 1.64)	*ANI*(6.06 ± 1.71)
8	*CYP*(5.18 ± 1.57)	*CYP*(6.04 ± 1.74)	*HSP70*(8.36 ± 2.62)	*CP*(4.51 ± 1.41)	*GADPH*(8.89 ± 1.97)	*CYP*(5.9 ± 1.73)
9	*ANI*(5.8 ± 1.75)	*ANI*(6.38 ± 1.74)	*50S*(8.22 ± 1.79)	*CYP*(5.39 ± 1.85)	*CYP*(6.85 ± 1.97)	*ACT*(5.91 ± 1.87)
10	*HSP70*(7.52 ± 2.32)	*SuS*(6.66 ± 2.03)	*ANI*(6.88 ± 1.94)	*ACT*(5.65 ± 1.81)	*HSP70*(7.36 ± 2.21)	*HSP70*(6.31 ± 1.99)
11	*50S*(9.44 ± 2.14)	*ACT*(6.91 ± 2.17)	*ACT*(6.9 ± 2.16)	*HSP70*(5.93 ± 1.91)	*50S*(11.55 ± 2.51)	*50S*(10.29 ± 2.24)
12	*GADPH*(10.35 ± 2.34)	*50S*(10.95 ± 2.31)	*H3*(9.05 ± 2.6)	*SuS*(7.42 ± 2.34)	*SuS*(8.47 ± 2.58)	*H3*(8.31 ± 2.38)
13	*SuS*(7.75 ± 2.47)	*H3*(8.85 ± 2.46)	*SuS*(9.95 ± 3.04)	*50S*(11.01 ± 2.4)	*H3*(11.59 ± 3.14)	*SuS*(8.35 ± 2.59)

**Table 2:** The expression stability values of candidate reference genes in centipedegrass were computed using NormFinder software.

Rank	Cold Stress	Salt Stress	Drought Stress	Al Stress	Herbicide Stress	All Samples
1	*UBC*(1.006)	*MD*(0.478)	*MD*(0.352)	*MD*(0.895)	*ANI*(0.701)	*MD*(0.941)
2	*MD*(1.230)	*RIP*(0.582)	*RIP*(0.753)	*UBC*(0.943)	*RIP*(0.914)	*RIP*(0.959)
3	*ANI*(1.270)	*ANI*(0.996)	*ADP*(0.855)	*RIP*(0.991)	*ADP*(1.067)	*ADP*(1.245)
4	*RIP*(1.308)	*UBC*(1.040)	*GADPH*(1.190)	*ADP*(1.080)	*ACT*(1.103)	*UBC*(1.323)
5	*CYP*(1.427)	*GADPH*(1.293)	*UBC*(1.616)	*ANI*(1.117)	*CP*(1.268)	*ANI*(1.339)
6	*ADP*(1.481)	*CYP*(1.353)	*CP*(1.772)	*CP*(1.192)	*MD*(1.298)	*CP*(1.541)
7	*CP*(1.489)	*CP*(1.551)	*ANI*(1.779)	*GADPH*(1.23)	*UBC*(1.747)	*CYP*(1.568)
8	*50S*(1.852)	*ADP*(1.566)	*CYP*(1.838)	*CYP*(1.345)	*CYP*(1.865)	*ACT*(1.865)
9	*ACT*(1.976)	*SuS*(1.636)	*ACT*(2.263)	*ACT*(1.773)	*GADPH*(2.272)	*GADPH*(2.304)
10	*H3*(2.198)	*ACT*(1.797)	*H3*(2.286)	*HSP70*(1.814)	*HSP70*(2.701)	*50S*(2.416)
11	*SuS*(3.178)	*50S*(2.149)	*50S*(2.998)	*H3*(1.991)	*50S*(2.741)	*H3*(2.468)
12	*GADPH*(3.555)	*H3*(2.294)	*SuS*(3.749)	*50S*(2.121)	*SuS*(2.851)	*HSP70*(2.527)
13	*HSP70*(4.490)	*HSP70*(2.362)	*HSP70*(5.39)	*SuS*(2.405)	*H3*(3.138)	*SuS*(2.839)

**Table 3:** The RefFinder analysis identified the combination of reference genes that exhibited the highest and lowest stability.

Method	**Stability (High–Low)**												
	1	2	3	4	5	6	7	8	9	10	11	12	13
**Cold stress**													
BestKeeper	*UBC*	*RIP*	*CP*	*H3*	*ADP*	*MD*	*ACT*	*CYP*	*ANI*	*HSP70*	*50S*	*GADPH*	*SuS*
NormFinder	*UBC*	*MD*	*ANI*	*RIP*	*CYP*	*ADP*	*CP*	*50S*	*ACT*	*H3*	*SuS*	*GADPH*	*HSP70*
Genorm	*UBC/MD*		*ANI*	*ADP*	*CP*	*RIP*	*CYP*	*ACT*	*H3*	*50S*	*HSP70*	*SuS*	*GADPH*
RefFinder	*UBC*	*MD*	*RIP*	*ANI*	*ADP*	*CP*	*CYP*	*H3*	*ACT*	*50S*	*SuS*	*GADPH*	*HSP70*
**Salt stress**													
BestKeeper	*MD*	*GADPH*	*ADP*	*UBC*	*RIP*	*CP*	*HSP70*	*CYP*	*ANI*	*SuS*	*ACT*	*50S*	*H3*
NormFinder	*MD*	*RIP*	*ANI*	*UBC*	*GADPH*	*CYP*	*CP*	*ADP*	*SuS*	*ACT*	*50S*	*H3*	*HSP70*
Genorm	*RIP/MD*		*ANI*	*UBC*	*ADP*	*GADPH*	*CYP*	*SuS*	*ACT*	*CP*	*H3*	*HSP70*	*50S*
RefFinder	*MD*	*RIP*	*UBC*	*GADPH*	*ANI*	*ADP*	*CYP*	*CP*	*SuS*	*ACT*	*HSP70*	*50S*	*H3*
**Drought stress**													
BestKeeper	*GADPH*	*MD*	*ADP*	*RIP*	*CYP*	*CP*	*UBC*	*HSP70*	*50S*	*ANI*	*ACT*	*H3*	*SuS*
NormFinder	*MD*	*RIP*	*ADP*	*GADPH*	*UBC*	*CP*	*ANI*	*CYP*	*ACT*	*H3*	*50S*	*SuS*	*HSP70*
Genorm	*RIP/MD*		*CP*	*CYP*	*HSP70*	*ADP*	*GADPH*	*UBC*	*ANI*	*ACT*	*H3*	*50S*	*SuS*
RefFinder	*MD*	*RIP*	*GADPH*	*ADP*	*UBC*	*CP*	*CYP*	*ANI*	*H3*	*ACT*	*50S*	*SuS*	*HSP70*
Method	**Stability (High–Low)**												
	1	2	3	4	5	6	7	8	9	10	11	12	13
**Al stress**													
BestKeeper	*RIP*	*GADPH*	*MD*	*UBC*	*ADP*	*ANI*	*H3*	*CP*	*CYP*	*ACT*	*HSP70*	*SuS*	*50S*
NormFinder	*MD*	*UBC*	*RIP*	*ADP*	*ANI*	*CP*	*GADPH*	*CYP*	*ACT*	*HSP70*	*H3*	*50S*	*SuS*
Genorm	*RIP/MD*		*ADP*	*UBC*	*ANI*	*GADPH*	*H3*	*CP*	*CYP*	*HSP70*	*ACT*	*SuS*	*50S*
RefFinder	*UBC*	*MD*	*RIP*	*GADPH*	*ADP*	*ANI*	*CP*	*CYP*	*H3*	*ACT*	*HSP70*	*50S*	*SuS*
**Herbicide stress**													
BestKeeper	*RIP*	*ADP*	*MD*	*UBC*	*ACT*	*ANI*	*CP*	*GADPH*	*CYP*	*HSP70*	*50S*	*SuS*	*H3*
NormFinder	*ANI*	*RIP*	*ADP*	*ACT*	*CP*	*MD*	*UBC*	*CYP*	*GADPH*	*HSP70*	*50S*	*SuS*	*H3*
Genorm	*ADP/RIP*		*ANI*	*ACT*	*CP*	*MD*	*UBC*	*CYP*	*GADPH*	*HSP70*	*SuS*	*H3*	*50S*
RefFinder	*RIP*	*ANI*	*ADP*	*ACT*	*MD*	*UBC*	*CP*	*GADPH*	*CYP*	*HSP70*	*50S*	*SuS*	*H3*
**All samples**													
BestKeeper	*MD*	*ADP*	*RIP*	*UBC*	*GADPH*	*CP*	*ANI*	*CYP*	*ACT*	*HSP70*	*50S*	*H3*	*SuS*
NormFinder	*MD*	*RIP*	*ADP*	*UBC*	*ANI*	*CP*	*CYP*	*ACT*	*GADPH*	*50S*	*H3*	*HSP70*	*SuS*
Genorm	*ADP/RIP*		*MD*	*UBC*	*ANI*	*CP*	*CYP*	*ACT*	*H3*	*HSP70*	*GADPH*	*SuS*	*50S*
RefFinder	*ANI*	*RIP*	*UBC*	*ADP*	*CYP*	*ACT*	*GADPH*	*MD*	*SuS*	*H3*	*50S*	*CP*	*HSP70*

## Data Availability

Not applicable.

## Supplementary Materials

The following supporting information can be downloaded at: https://www.mdpi.com/article/10.3390/genes14101874/s1, Table S1. Description of 13 reference genes and 5 target genes; Figure S1. Primer specificity of 13 candidate reference genes and 5 target genes.
