# Supplementary material for: Selection of Suitable Reference Genes for RT-qPCR Gene Expression Analysis in Centipedegrass under Different Abiotic Stress
Source: Genes (Basel). 2023 Sep 26;14(10):1874. doi: 10.3390/genes14101874 (PMC10606319; doi:10.3390/genes14101874)
Supplement: Supplementary file 1 [file genes-14-01874-s001.zip › genes-2617471-supplementary.pdf]

Table S1 Description of 13 reference genes and 5 target genes.

Gene Symbol	Gene Name	ID	Primer: Forward/Reverse	Amplification Product Size (bp)
UBC	*Ubiquitin-conjugating enzyme*	D_transcript_97520	GCAATGGGCTGACAAATACC<br>AGGCTGACATCGACTACAATAC	153
GADPH	*Glyceraldehyde-3-phosphate dehydrogenase*	D_transcript_100518	GGTGTACGAAGCATGAGGTATT<br>CGGAAGCATGTACGAGTTGA	180
ACT	*Actin*	D_transcript_121418	TGCCTATGTTGCCCTTGATTA<br>TGAAGGATGGCTGGAATAGAAC	152
SuS	*sucrose synthase*	D_transcript_120900	GGTACTGAGCACACAGACATTA<br>CTAGGTTGCCATCACTGTAGTT	190
ANI	*Alkaline and neutral invertase*	D_transcript_129215	CAGATGACAAGTGGCCTGAATA<br>CTCATCGCAGGTCAGAATAGAAG	155
ADP	*ADP-ribosylation factor*	D_transcript_105677	GAACAAGCCGAGTACCTCAATA<br>GTTCAAACGCCCTGTCAATATG	177
CYP	*Cyclophilin*	D_transcript_18371	GTCGAAGCTATGATCTGGTCTTT<br>GTGTGCCCTCTCATGTCTATTT	161
H3	*Histone H3*	D_transcript_105356	AAACCGCTCTTCGCTGATT<br>GGTAACGGTGAGGCTTCTTT	198
50S	*50S ribosomal protein L2*	D_transcript_1908	CCGACCTGGCTTACGAATTTA<br>GTACTATAGGTGCGGCCATTAC	175
RIP	*60S ribosomal protein L2*	D_transcript_24803	GGAACATGGGTGCATGATATTG<br>GGCTTGGATTGGAGACTATACC	187

Gene Symbol	Gene Name	ID	Primer: Forward/Reverse	Amplification Product Size (bp)
MD	*Malate dehydrogenase*	D_transcript_49239	CTCTGATCCATCGAGTCACATTAC<br>GAGGGAAGCTCTGATGTTCATT	174
CP	*Chaperone protein*	D_transcript_47760	GGCGAAAGCCTACTGGATAAA<br>CCTCCAGATTCTCCTTGATTGG	169
HSP70	*Heat shock 70 kDa protein*	D_transcript_6384	CAACAACAGCACTGCCTTTAG<br>GAGTTCTACCCTGCACGATTT	164
PIP1	*Phosphatidylinositol phosphate*	D_transcript_52676	CATCTCCGAGGACACATCAA<br>CCACCATTGCCCATGTAAAG	168
PAL	*Phenylalanineammonia-lyase*	D_transcript_14142	GCAACTTCCAGGGCACCC<br>CTCCGAGAACTGAGCGAACAT	151
Cor413	*Cold-Regulated 413*	D_transcript_27921	TCTATTCTTTCCACGCCACTTC<br>CCTGACGCCTTGATATGTTCTT	162
ALMT9	*Aluminum-activated malate transporters 9*	D_transcript_99202	ATCCGAAGATGAAGCCGTATG<br>CCAGATTGGGTAGATGCCTATATT	174
BAR	*BAR*	D_transcript_33677	CAAGTGCCCGTATAGATGGATTAG<br>CATACACATGCCTCCCTTTCA	183

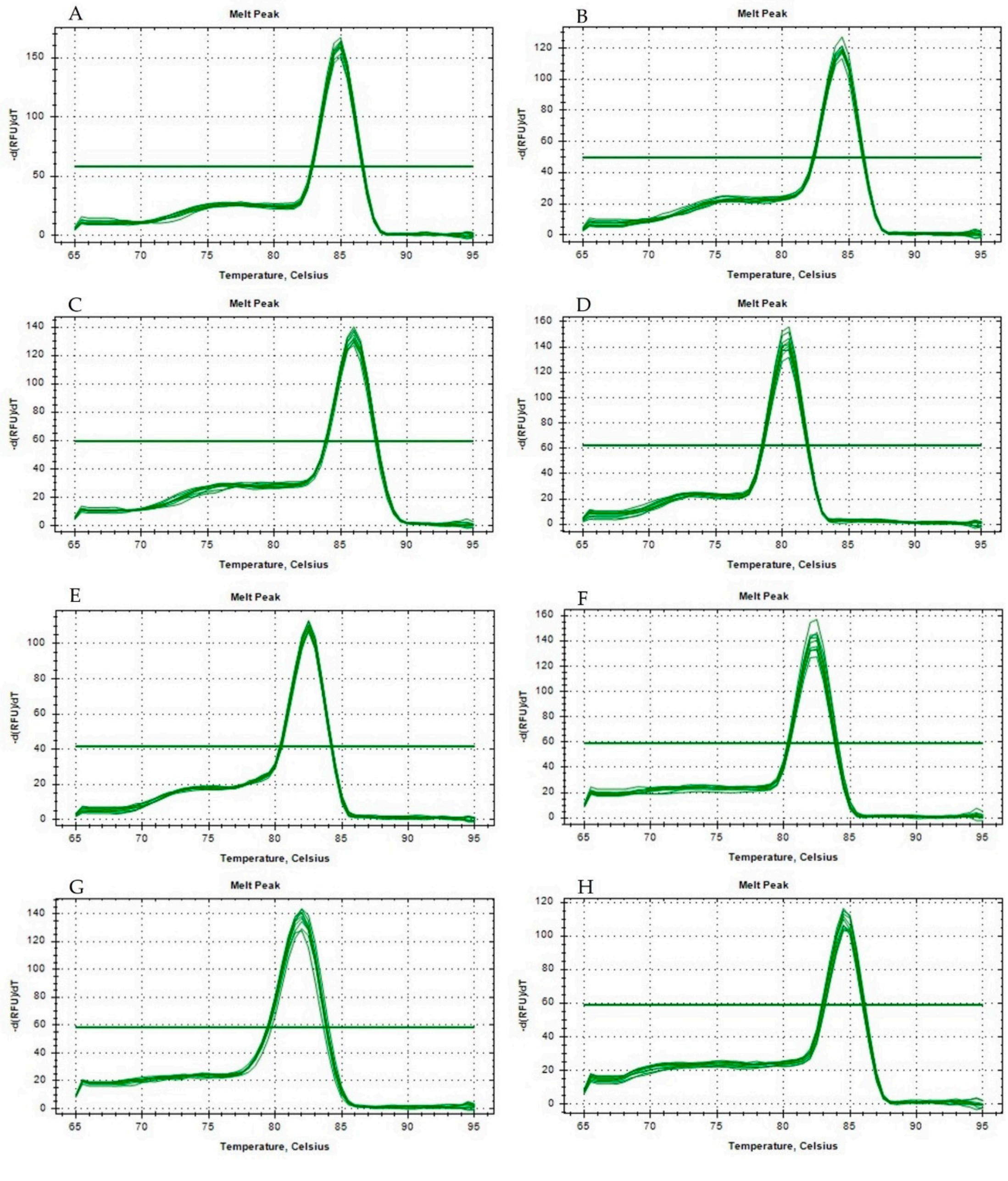

A
Melt Peak
-d(RFU)/dT
Temperature, Celsius
B
Melt Peak
-d(RFU)/dT
Temperature, Celsius
C
Melt Peak
-d(RFU)/dT
Temperature, Celsius
D
Melt Peak
-d(RFU)/dT
Temperature, Celsius
E
Melt Peak
-d(RFU)/dT
Temperature, Celsius
F
Melt Peak
-d(RFU)/dT
Temperature, Celsius
G
Melt Peak
-d(RFU)/dT
Temperature, Celsius
H
Melt Peak
-d(RFU)/dT
Temperature, Celsius

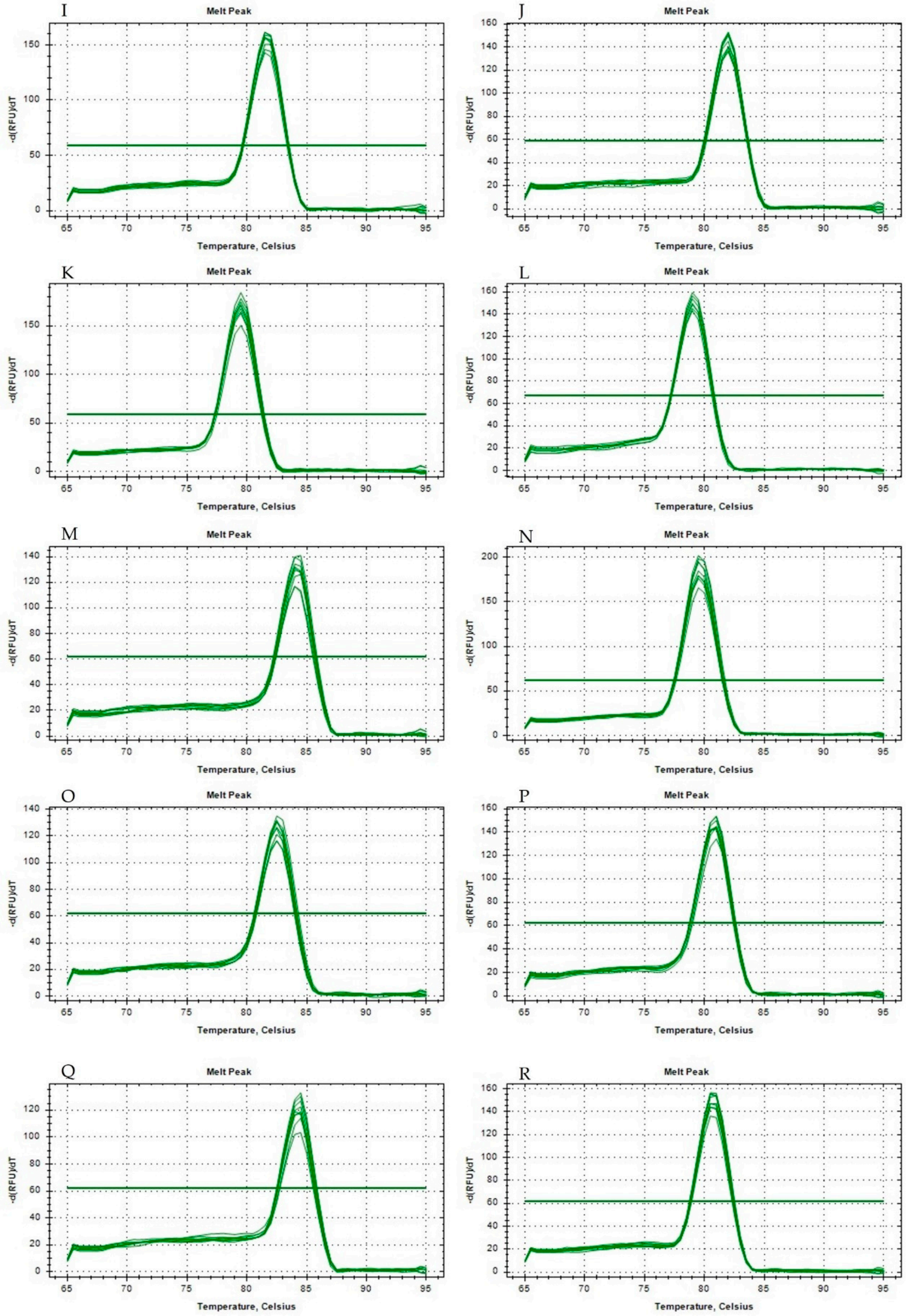

Figure S1 Primer specificity of 13 candidate reference genes and 5 target genes. (**A**) *PIP1*; (**B**) *COR413*; (**C**) *PAL*; (**D**) *ALMT9*; (**E**) *BAR*; (**F**) *UBC*; (**G**) *GADPH*; (**H**) *ACT*; (**I**)*SuS*; (**J**) *ANI*; (**K**) *ADP*; (**L**) *CYP*; (**M**) *H3*; (**N**) *50S*; (**O**) *RIP*; (**P**) *MD*; (**Q**) *CP*; (**R**) *HSP70*.
